# Facilitating health and wellbeing is “everybody’s role”: youth perspectives from Vanuatu on health and the post-2015 sustainable development goal agenda

**DOI:** 10.1186/s12939-014-0080-8

**Published:** 2014-10-10

**Authors:** Simon A Sheridan, Claire E Brolan, Lisa Fitzgerald, John Tasserei, Marie-France Maleb, Jean-Jacques Rory, Peter S Hill

**Affiliations:** School of Population Health, The University of Queensland, Brisbane, Australia; Health Promotion Unit, Public Health Department, Port Vila, Efate Vanuatu; Sanma Provincial Health Office, Luganville, Espiritu Santo Vanuatu

**Keywords:** Vanuatu, Youth, Young people, Post-2015 agenda, Millennium development goals, Sustainable development goals, Health policy

## Abstract

**Background:**

Progress towards achievement of the Millennium Development Goals (MDGs) amongst Pacific island countries (PICs) has seen mixed results. As focus shifts to formulation of new health-related development goals beyond 2015, there is a need for bringing community consultation into this process. For this purpose, *Go4Health* is a global consortium examining the development of these goals, with *Work Package 2* capturing viewpoints of marginalised populations regarding health. This paper examines the perspectives of youth in Vanuatu on essential health needs in the context of the post-2015 development agenda, to make these concerns more visible for their communities, stakeholders and health policy decision makers.

**Methods:**

As part of a larger investigation undertaken in Vanuatu involving 100 residents from various rural and urban communities, this paper explores the perspectives of twenty 17-year old secondary school students gathered through two focus group discussions during September 2013. Questions sought viewpoints across areas including health ideals, essential needs, responsibility for health services and their governance. Focus group discussions were conducted in English and digitally recorded, with resulting transcripts subjected to thematic analysis.

**Results:**

This youth cohort from Vanuatu had a strong understanding of the social determinants of health. They placed value on all aspects of health, indicating the need for youth to have access to positive lifestyle opportunities (sport, community participation) and also increased protection from the impact of harmful substances and causes of chronic illness. Participants identified barriers to health due to unevenly distributed health services throughout Vanuatu, with members at all levels of society ultimately perceived as responsible for improving health throughout the nation.

**Conclusion:**

Against a background of a weak health system and significant challenges to public health, young people are acutely aware that improving Vanuatu’s health status requires a communal effort. While contributing factors to health depend on actions taken at individual, local, national and global levels, no single actor currently provides enough support to cover all essential health needs. As a consequence, they see health in the Pacific as “everybody’s role”, of importance for the post-2015 sustainable development goal agenda and health policy makers in general.

## Introduction

Progress towards achievement of the Millennium Development Goals (MDGs) by 2015 has undoubtedly contributed to improving the health status of countless people worldwide [1]. Meanwhile, across the Pacific island countries (PICs) and other regions, there have been mixed results, with many countries falling short of the globally set health targets [2,3]. That incomplete agenda will be carried forward in the formulation of a new sustainable development goal agenda to replace the MDGs, after they expire in 2015.

While the MDG process has proved a useful driver for development, the process of their creation gave no direct say to those who face the most pressing health issues in low-to-middle income countries (LMICs) [4-6]. The new sustainable development goals will be universal, but set differentiated targets that are linked to each country’s unique context and needs. To successfully achieve this, community consultation and involvement must be integral to this process [7]. While the consultation process has been extensive, with a number of high level forums with representatives from many different countries providing valuable input for the new agenda, and a web based consultation inviting global responses, concerns remain around the inequitability of access to such global discussion forums [8].

*Go4Health* is a global consortium of academic, civil society and non-government organisations examining the evolution of the post-2015 sustainable development agenda, with a particular focus on health. It has tasked *Work Package 2* with capturing viewpoints of vulnerable or marginalised populations regarding their own health needs, for inclusion in debate around the sustainable development goals [9]. Without a particular focus on addressing the health needs of the most disadvantaged within any given society, such people tend to miss out on sharing in the health benefits experienced by the wider population, as data from many settings has shown [10]. With this in mind, disadvantaged communities from a number of regions have been selected for study in *Go4Health’s Work Package 2*, including Vanuatu in the Pacific region.

In this study, undertaken for the *Go4Health* project, the voices of Pacific youth were engaged as part of a qualitative investigation conducted throughout Vanuatu, which is classified as a lower-middle income country by the World Bank [11]. The rationale for selection of a cohort of youth for inclusion in this project was based on the idea that youth can provide insights relevant for a community’s needs over the long term, just as this generation enters its active reproductive and working years. Furthermore, emerging global public health challenges, including non-communicable diseases (NCDs), are often related to lifestyle conditions that begin to develop during the second decade of human life [12].

Consequently, there is increased focus on prioritizing the health of the world’s 1.8 billion young people, of which 90 % live in low-income countries [13-16]. Young people are at the centre of major global social and environmental issues including youth unemployment, urbanisation, migration, unrest and conflict, which influence health and wellbeing [12,17,18]. However, most research on young people’s health is focused on so-called “risk” behaviours, often dislocated from context [19]. Moreover, knowledge of young people’s health is particularly limited outside of high income countries [14]. The consideration of Pacific youth health concerns for the post-2015 development agenda and beyond is therefore of great importance [17].

Indeed, there has been growing concern for grounding the new post-2015 development agenda in an approach that supports a healthy life throughout the life course of an individual [12,17,18]. In May 2013, the *High-Level Panel of Eminent Persons’ Report* on the agenda identified the concerns of young people as a cross-cutting issue. It stated that “young people must be subjects, not objects” of the agenda [20]. The panel also acknowledged a key health concern raised by young people was their ability “to be able to make informed decisions about their health and bodies, to fully realize their sexual and reproductive health and rights” [20].

The aims of this qualitative study are twofold. First, to identify the perceptions of young people in Vanuatu regarding gaps in coverage of essential health needs, including those experienced by their peers and others within their communities. Second, to explore potential issues involved in meeting those needs. Through a process of consultation with young people to determine local and regional health issues pertinent to this generation, this study also intends to make their concerns more visible for their own communities, as well as for stakeholders and decision makers involved in health policy [16].

## Background

**Figure 1:**
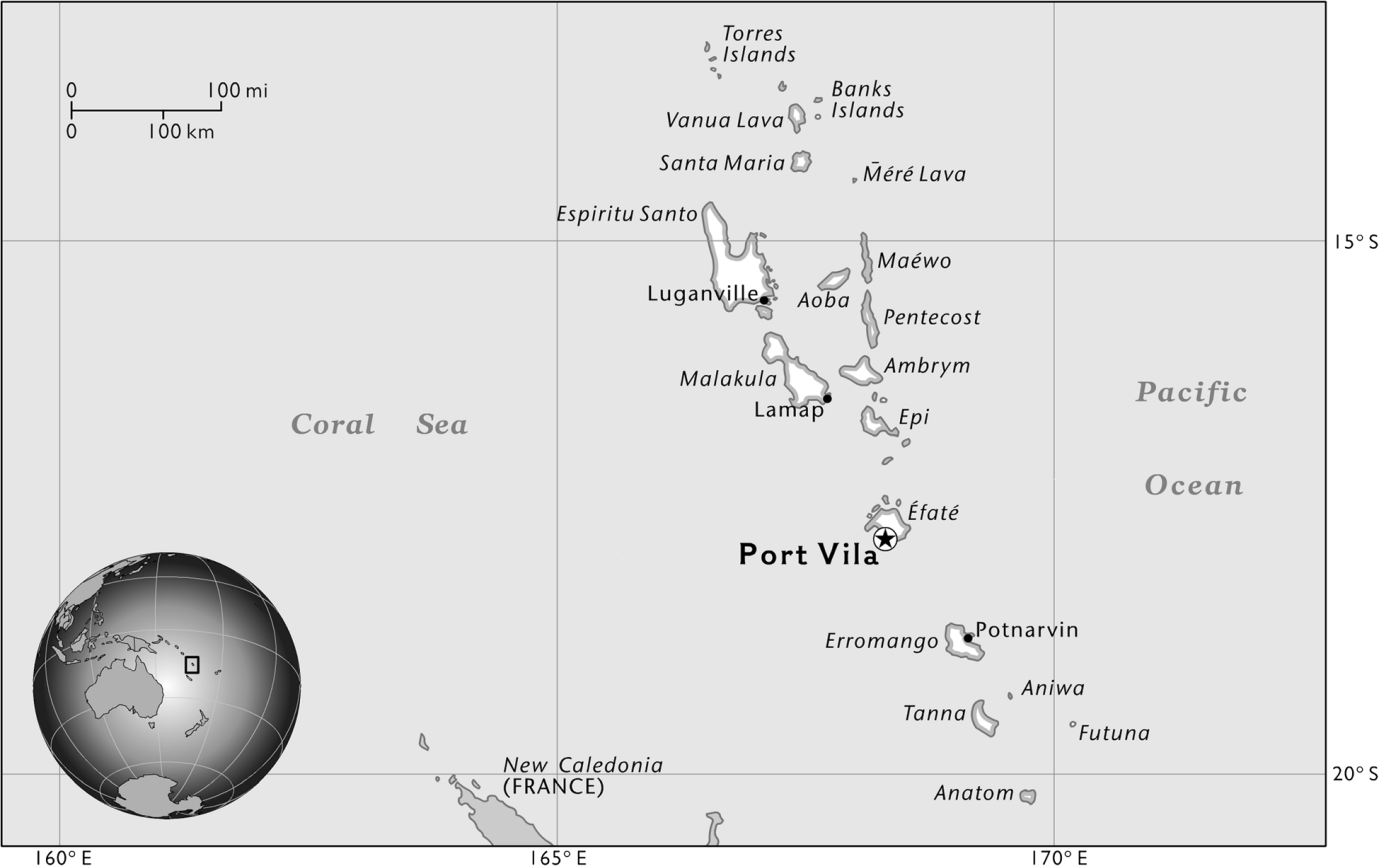
**Map showing the island archipelago of Vanuatu [** 24 **].**

Vanuatu’s population of around 260,000 is spread throughout an archipelago of 83 islands (Figure 1), resulting in geographic isolation for many small communities distantly located from the major urban centres. Of the national population, approximately 19 % is aged 15–24 years, which is how the UN defines youth [21,22]. As post-primary schooling is not compulsory, the employment sector is under pressure to absorb those who discontinue formal education early [22]. Indeed, limited opportunities exist for youth to participate fully in their communities, due to the effects of both stagnating economies and the conservative, patriarchal structures of Pacific societies [23].

Rapid population growth In Vanuatu is being driven by one of the highest national fertility rates in the Pacific region, and few policies are currently in place to cope effectively with the increasing youth bulge [25]. In fact, efforts to promote condom use amongst youth in both Vanuatu and Tonga were found to be inefficient without taking social and cultural barriers into account [26], with surveys revealing relatively low contraceptive use (34.5 %) amongst teens [27-29]. While incomplete data collection hampers accurate tracking of unintended pregnancy and sexually transmitted illnesses among adolescents [30,31], there is virtually no reliable data to estimate the prevalence of HIV/AIDS in Vanuatu [32]. Meanwhile, studies have shown that unmet needs for family planning persist due to low uptake of reproductive health services by youth in Vanuatu, attributable to factors such as shame, lack of awareness and judgemental attitudes by service providers [33].

However, in recent years adolescent girls (aged 15–19 years) have increased their utilisation of antenatal care (84.9 %) and skilled personnel to assist during birth (72.9 %) [29]. This relates to recent estimates which indicate that maternal mortality has been halved since 1990, despite a lack of reliable tracking data to confirm further progress [29,32]. Vanuatu’s rate of under-five child mortality per 1000 live births has successfully decreased from 58 in 1990, to 30 in 2007 and is potentially on track to meet the target of only 19 by 2015. Also, immunisation of 1 year-olds against measles increased from 66 % coverage in 1990 to 80 % in 2009, with the 2015 target of 95 % still within reach [34].

Despite Vanuatu’s progress with the health-related MDGs, non-communicable diseases (NCDs) persist as a major cause of death, despite poor data. In 2002, an estimated 800 of a total of 1220 recorded deaths were attributed to NCDs [35], with more recently published reports sustaining this estimate [36]. Studies have also explored factors that influence youth to adopt lifestyles related to the development of chronic illness. While one study of Pacific youth has attempted to explore the link between positive emotional state and avoidance of tobacco smoking [37], most studies in this region investigated the negative effects of the prevalence of adolescent tobacco, cannabis and injecting drug use [38-40] as well as degree of self-harm and bullying amongst youth in several PICs [41,42].

Other research on Pacific youth health has generated biometric data. This includes anthropometric surveys to gauge the effect of various levels of economic development on childhood and adolescent overweight and obesity throughout Vanuatu [43], and results from the health and lifestyle surveys conducted during 2000–01. These reported on levels of substance abuse, physical activity, dietary choices and social interaction of over 4500 young people in Vanuatu [44,45] and may be useful for tracking trends over time. Overall, published studies of Pacific youth health mainly focus on “risk” behaviours, rather than framing youth health in a more empowering and comprehensive way that has been advocated elsewhere [17]. Hence, this paper provides valuable insights regarding Pacific youth health.

## Methods

### Setting

Vanuatu’s main industries include agriculture, tourism and financial services, but the country is largely dependent on foreign aid, especially for large scale infrastructure projects [46]. Its population is largely rural (74 %), relying on subsistence farming and fishing [47]. Outside urban areas, literacy is low [48] and use of traditional healers and natural bush medicine is common [49]. There are three official national languages (Bislama, English and French) and at least 80 indigenous languages [48] used throughout the archipelago.

As part of the *Go4Health* project, a qualitative study of essential health needs was undertaken throughout Vanuatu between September 2–17, 2013. This was a joint effort by researchers from the University of Queensland and the Vanuatu Ministry of Health. Overall, participants were drawn from a diverse range of communities involving both adolescent and adult members (n = 100). To provide a representation of the national *ni-Vanuatu* (local or indigenous) population, the overall study sought to include residents from a mix of urban, rural and remote communities, located on three different islands: Efate (located centrally in the Vanuatu island archipelago, home to the capital of Port Vila), Espiritu Santo (to the north of Efate), and Tanna (in the south). While communities were selected in order to reflect this geographic spread, they were ultimately included on the basis of timely access and being granted permission by the village chief or senior leaders in each community. Table 1 summarizes the characteristics of each focus group discussion, including location, number, gender and type of participants, as well as the language used.

**Table 1:** **Summary of focus group discussions and participants involved in the overall study conducted throughout Vanuatu**

**Focus group no.**	**Location, name of island**	**Number of participants**			**Participant type**	**Language spoken**
		**Total**	**Female**	**Male**		
1	Urban, Efate	n = 10	n = 5	n = 5	Secondary school students	English
2	Rural, Efate	n = 12	n = 6	n = 6	Parents of primary school children	Bislama
3	Rural, Efate	n = 10	n = 4	n = 6	Adult community members	Bislama
4	Remote, Tanna	n = 10	n = 5	n = 5	Adult villagers	Bislama
5	Remote, Tanna	n = 10	n = 5	n = 5	Adult villagers	Bislama
6	Rural, Tanna	n = 8	n = 1	n = 7	Adult community members	English, Bislama
7	Remote, Espiritu Santo	n = 11	n = 5	n = 6	Adult villagers	Bislama
8	Rural, Espiritu Santo	n = 11	n = 6	n = 5	Adult community members	Bislama
9	Urban, Espiritu Santo	n = 8	n = 6	n = 2	Parents of primary school children	Bislama
10	Urban, Espiritu Santo	n = 10	n = 5	n = 5	Secondary school students	English
Total		n = 100	n = 48	n = 52		

### Data collection

The present paper reports on a discrete study of a group of adolescents (n = 20), which formed part of the larger, overall study mentioned above. In order to gain access to a sample of adolescents from various islands, boarding schools were contacted, at which students were drawn from urban, rural and remote locations throughout Vanuatu. To recruit focus group participants, purposive sampling methods [50] were employed; that is, school principals were requested to include students based on demonstration of leadership ability and not upon demonstration of academic ability alone, in order to select for participants confident in expressing their opinions on community matters.

Secondary school students participated in two English language focus group discussions (one in Efate, one in Espiritu Santo). Each group comprised five male and five female adolescents (all aged 17 years) in their final year of high-school. A range of community types was represented: of 20 participants in total, 16 were from rural and remote locations, and 4 from urban locations. Also, a range of geographical locations was represented: participants identified as originating from 11 of Vanuatu’s 83 islands.

Using a question guide developed and tested previously in several international settings by members of the *Go4Health* consortium [8,9], five broad domains of inquiry were discussed with participants: (i) how young people define health; (ii) social determinants of health; (iii) community perceptions of essential health needs; (iv) roles and responsibilities of relevant actors in health; and (v) community participation in decision-making.

The focus group discussions were conducted in private, without teachers or relatives of participants present, so that they could speak freely; participants were informed that all possible answers were valid; they were also encouraged to give preference to their personal opinions and speak about their own experiences.

### Data analysis

Focus group discussions were digitally recorded and transcribed. A deductive thematic analysis of the transcripts was performed [51], and data was coded according to emerging categories. Quotations were grouped into four categories. Findings were corroborated through discussions with a number of staff within Vanuatu’s Ministry of Health.

### Ethical considerations

Ethics approval was obtained through the University of Queensland School of Population Health’s Human Research Ethics Committee, and in-country research was authorized by the director of Public Health at Vanuatu’s Ministry of Health. Informed consent was provided by all students involved in the focus group discussions and their participation was authorised by the school principals as their delegated guardians. Participants were informed of their ability to withdraw at any time, and that their responses would be de-identified for privacy.

## Results

The essential health needs identified by young people in Vanuatu are represented by four main themes: (1) youth definitions of ideal health; (2) unhealthy lifestyle choices; (3) health worker shortage; and (4) responsibility for achieving good health.

### Theme 1: Youth definitions of ideal health

Participants had a comprehensive understanding of the multi-dimensional nature of health, and raised three elements that comprised good health and well-being. The first element, particularly raised by males within both focus groups, was the importance of physical health, of a sense of energy, physical fitness, and strong physical appearance. Responses included references to physical fitness and activity, body shape, and sport. The second element of good health identified by both male and female respondents was psychological health and well-being. Well-being was not only defined as the absence of worry and stress; importantly, having a positive self-image, a sense of self-worth, and thinking positively were identified as necessary components. One female participant explained:

*“[Something] which helps keep a person healthy is positive thinking…being confident in yourself…because health is not only keeping physically fit, it’s also your mind, and dealing with your mind and everything like that.”*

Another female participant stated:

*“My definition of health would be having a sense of being satisfied with myself, in terms of how well I do my work, or how I communicate with others, and also how I enjoy eating my food. For me, health is more like being satisfied with everything that I do.”*

In addition, many participants identified and placed value on the underlying social determinants of health, which emerged as the third element of health. Access to clean water and nutritious food, sanitation, proper housing, a clean environment, and the population pressures caused by rural to urban migration, were all mentioned. For example, one female participant described how people lived in her village:

*“They don’t have good homes, don’t have a proper disposal system for rubbish. So they dump their rubbish into the river or along the sides of the roads. That does affect the health of the people, because sometimes the children climb over the rubbish, collect stuff like bottles to sell. Everything you can find is in that rubbish. From plastics to tins, and food rubbish, diapers, and even sewerage. All these things, they just dump them along the roadsides, or in the water. So, it really does affect the health of the people around the area. You can tell by their children, looking skinny, and having pot bellies, and yet so skinny.”*

Furthermore, in relation to maternal and child health, a female participant placed value on community support structures that help young women to raise children:

*“Nowadays in Vanuatu, young girls, teenage pregnancy is very high.. [they need advice] to look after their children, bring them up in a proper healthy way… [including] using local foods, training their taste buds and stuff like that…simple things that are contributing to our health.”*

Several participants expressed understandings of health that were not only individual but also communal: many participants observed the causative and interconnected nature of social determinants on the overall level of health experienced by their home village or community.

### Theme 2: Unhealthy lifestyle choices

Access to psychoactive substances including kava and marijuana was identified by participants as having a negative impact on their community’s health. Some only discussed substance abuse among their parents’ generation, and their exposure to this. For example, one youth was determined not to feel bad like his father, who indulged in drinking kava, smoking and drugs. Another participant spoke of recurring drug abuse in her community, including the use of alcohol by her uncles. The common misuse of kava was identified:

*“I mean, nowadays, we do have access to alcoholic drinks but before…[and] still going on very highly…is the drinking of kava. This is very high…amongst males. And some females too, a lot of females, nowadays yes. They drink a lot of kava…families, they just mix it up and they drink it nearby the house. If not, usually, daily, weekdays, the fathers and the mothers, they just go to the kava bars.”*

Several other participants highlighted the prevalence of substance abuse among the nation’s young people. One female participant spoke of her concern that young people in Vanuatu were taking up drugs across a number of islands. These participants were concerned that young men, in particular, were engaging in substance abuse.

*“Also, alcohol and stuff like that, and home-brew. It’s not good for your health. In the islands we don’t have shops and stuff like that. So the boys, especially the boys, they like to prepare their own home-brew…the boys in the villages use yeast, they mix it up with sugar.”*

*“And for young people, boys especially, at night they would hang around the road, they get involved in drugs and stuff, marijuana.”*

One participant observed that relaxed alcohol sales practices were resulting in young people’s ability to access low-cost alcoholic drinks, with some shop owners turning a blind eye to the age of the purchaser. This participant also noted such drinks were potentially dangerous:

*“What you find is, like if it’s a party where there’s alcohol, you’d find most of the time cheap alcohol [present]. Well, the shops nowadays, the Chinese shops, they’re selling it. It’s called “Nine-fifty” because it’s 950 Vatus. About ten dollars for a bottle. And rum, vodka and stuff…[Q: What’s the age you have to be to buy alcohol from shops?] Eighteen, but they don’t care.. [Q: Who?] The Chinese shops. All the shops but mostly the Chinese shops…Well if you went to a shop owned by a local, sometimes, they would know you…that you’re underage, and they wouldn’t sell you the drink. But I found out that these “Nine-fifty” drinks, they’re actually just plain water, mixed with pure alcohol. And they just put the flavour of the rum or vodka in, and then the Chinese are selling it. So it’s very unhealthy.”*

Participants also recognised the impact of globalization and the importation of cheap packaged food items contributed to poor health in the community, as the low-cost of such food was pricing out locally grown healthy food sources.

*“In Vanuatu, there are more products, I mean we have a lot of [food imports] that are produced overseas and they’re not that healthy compared to our local grown crops that are only on our islands. There are substances and chemicals that are put in [the imported food] and in Vanuatu we are not that well educated to know what they contain…It’s become a habit for us to [consume these imported products], because local grown foods are quite expensive compared to products that are imported into our country.”*

The above participant also acknowledged that the community’s lack of knowledge about the content of these imported food items compounded the issue. As the following participants note:

*“In Vanuatu there is importation of cheap food. So those cheap foods contain a lot of saturated fats and other stuff that’s not too healthy for you. People in Vanuatu they don’t do that [check food labels and saturated fat content]. So, people end up buying the cheapest foods, which contain a lot of bad stuff.”*

*“Some people in the islands eat anything…Not all of us, but that’s what you see…Anything that comes their way that is edible, they eat it. Sometimes we don’t actually know that are we are spoiling ourselves, by eating this type of food.”*

The need for health awareness raising programs around balanced nutritional intake was also mentioned, as well as health education programs that catered to the needs of the local community. One student commented that in her village, some of the people did not have access to meat but only consumed taro, and sometimes they became sick by repeatedly eating the same food. As one female participant explained:

*“In these [remote] areas, they’re not that fortunate. I think through awareness programs and workshops, they will know how to look after themselves, in terms of eating habits. How are they supposed to know what is not good for them? Through awareness programs the health workers are able to pass on the message. Back in the village.. they learn there is another way.”*

One female participant expressed concern that her parents neither exercised, nor controlled their food intake, resulting in obesity. Alternatively, a male participant explained that considerable health promotion by government representatives was occurring throughout the country, to encourage physical exercise; however most people were neglecting this. Furthermore, this same participant expressed concern that local village leaders were often not reinforcing the government’s health promotion activities, and were seen to disregard the importance of physical exercise. In regard to sexual health, other participants recommended the need for public awareness around the consequences of unprotected sex with multiple partners. In this way, a male participant considered that awareness about HIV/AIDS was particularly lacking in Vanuatu’s remote highland areas:

*“They don’t know what AIDS is.. they think [unprotected sex] is fun for them.. but they can get sick.”*

Overall, participants were keen to acknowledge the impact of health education and its significance on lifelong health and safety.

### Theme 3: Health worker shortage

In terms of delivering good healthcare across the country, several participants recognised the issues and barriers were numerous and interconnected. The following female participant, for example, spoke about how health services were particularly lacking in rural and remote island areas. This participant elaborated how faulty equipment, poor medical facilities, a lack of technology and qualified medical staff all contributed to a deficit in services to and for the local people:

*“I think one of the needs for the communities is to upgrade the health system: hospitals, clinics, aid posts, and also to have equipment upgraded that is used there.. We also need to have qualified health workers, because in the islands we have locals who have just a little bit of understanding of how to treat just some sicknesses and basic knowledge on hygiene and how to keep themselves healthy. But we don’t have qualified nurses and doctors on all the islands of Vanuatu. Let me give an example - in the islands we don’t have microscopes and labs, and so when someone has malaria, sometimes it takes the doctors and nurses too long to find out.. and sometimes when they treat them it’s for a different sickness, when they really have malaria but are treated for something else…so we need to upgrade the systems and equipment so that we can have doctors to go down there and help the community.”*

A male participant then spoke specifically on health systems challenges due to lack of trained health staff, especially doctors:

*“We need to have, to have essential health that is…if you look at the number of doctors here [versus the number of citizens] …the doctors are [few in number]…when you compare with other countries, such as Australia…Even if you look at the hospital in Port Vila, the best facility around the country, you will see people, hundreds of people waiting for one doctor.”*

Additionally, the need for young people to be treated with respect by health care workers and in health care settings was recognized as particularly important. One female participant commented that a number of students had observed some clinic nurses and doctors interacting with patients in an impolite and rude manner; she suggested that this type of treatment did not help to make patients feel better. This participant expanded:

*“I mean, it’s very important in order for them to treat someone nicely so that they feel secure before they are treated. I’ll give you an example. My [relative], she’s a teenager, and its turned out she’s a victim of teen pregnancy, and she’s gone into labour. She was about to deliver and the nurses at the maternity ward threw her some nasty words. Through her labour she’s feeling pain, they’re just adding burdens to her…saying things like ‘why did you do that [fall pregnant]’, ‘that’s the problem with you young girls.’ In a way, it’s really none of their business, you’re put there to do a job, you’re not put there to interrogate girls or sick patients. I mean, you are there to encourage them, not to destroy their life with words or being rude.”*

There was acknowledgement that health workers were often stressed due to staff shortages, and were dealing with difficult situations:

*“They’re probably doing the best they can.. but it’s probably not good enough, because not everyone is getting their basic needs met.”*

### Theme 4: Responsibility for achieving good health

Within the focus group discussions, there was a strong sense that everybody had a duty to look after their own health, especially in light of poor health service coverage. Here, the participants are implicitly placing weight on the principle of individual agency in determining health outcomes. One female participant expands:

*“I think it’s the person who must be responsible. For example, I must be responsible to have a good education, and to make things to become realised. And to help my family and my community to achieve what they’re supposed to have…”*

*“I think it’s everybody’s role, because your health depends upon yourself, how you take care of yourself.”*

One male participant spoke of the responsibility especially resting on the shoulders of Vanuatu’s young people, the nation’s future:

*“The responsibility [is in the hands] of the young people. Youth should be taking the responsibility of the essential health needs of the community, as we determine the country’s future, young people…everyone, students, families, schools.”*

In contrast, two students (one male and the other female) considered the responsibility for good health rested not only on the individual, but multi-layered structural elements as well, which needed to work in partnership:

*“I think that responsibility for good health for our village, and our community, and our country, firstly is the government of the country, particularly the Minister of Health. Secondly, the chief of the villages, and thirdly, we must be responsible for ourselves. [For example] I must not drop litter in the waters…so that’s the reason why each of us must be responsible.”*

*“There are two groups of people that are the most important ones that are responsible for Vanuatu’s health. Firstly, it is the individuals…You yourself, what you eat, how you exercise. Secondly, the government. The two have to work together, yes…to provide facilities, proper health facilities…these two groups are the most important ones.”*

Other participants solely pointed to the role of the government, mainly the health department.

*“I think it’s the responsibility of the Department of Health… [to have enough medical facilities] on each island. To understand what are the needs of the people. Also, if people have needs, they can help search for them [for example]; if the population has a question about obtaining more medicines, they can look to the Department of Health for help.”*

*“What contributes to a person being healthy is the services that our government provides. For example, providing health services to the rural areas.”*

Alternatively, a number of participants recognised that the improvement of their country’s health and well-being was a complex and multifaceted issue. In fact, two male participants described it as being beyond the resource and financial capability of Vanuatu’s government and people, and tacitly beyond the prism of an in-country structure and agency debate.

*“..Because Vanuatu doesn’t have the funds to do all these things,so we obviously need aid, and then from this aid we should havequalified participants so that they can manage all these thingsproperly.”*

*“I think there’s a simple five letter word, and it’s called money. So for ourselves, our country - we cannot do that. I think we need aid from other countries, talking about things like a hospital… so now that’s why the Japanese government is trying to build our hospital up there. So I believe we can improve this in the near future.”*

For these participants, lasting measures to achieve a desirable level of health throughout Vanuatu’s islands was a matter of global concern, and depended upon the international community to share the responsibility.

## Discussion

The young people consulted for this study identified a wide range of unmet health needs in communities across Vanuatu. According to analysis of the results, the young people also placed value equally upon the physical, psychological and social aspects of health and well-being, which points to the need for Vanuatu’s youth to have access to positive opportunities for recreation such as sport, creative self-expression or meaningful community involvement. This is consistent with other research which suggests that a comprehensive, intersectoral approach is necessary to assist citizens to access ways to achieve and maintain good health that are culturally acceptable and context appropriate [52,53].

Participants in this study had a solid understanding of the influence of environmental and social determinants upon not just their own health, but also on the health of their entire community. By contrast, in resource-rich countries, qualitative studies on young people’s understandings of health repeatedly found that participants linked good health to individual or agency factors, such as risk behaviours, with minimal regard for the community-wide impact of social determinants of health [19,54,55]. Importantly, this disparity in level of community awareness, between youth from different resource settings, constitutes evidence of the varying sense of vulnerability experienced by individuals regarding control over their ability to stay healthy.

The widespread occurrence of substance misuse and adoption of unhealthy lifestyle practices, combined with the relative absence of protective measures for youth in Vanuatu appear to contribute to this sense of vulnerability. For example, while legislation is in place restricting the sale of alcohol to persons aged 18 years and over throughout Vanuatu [56], it appears that neither this, nor controls over quality of a bottle’s contents are strictly enforced. In addition to this, cultural acceptance of kava consumption and occurrence of intergenerational drug use may indicate that few safety measures are in place for youth that either prevent or delay undue exposure to harmful substances.

Indeed, health promotion activities by the health department were valued highly by participants and cited as an important method for educating the public. However, public health messages are competing with stark economic realities. Data across the Pacific confirms in recent years the affordability of locally produced food has decreased in light of the increased availability of cheaply imported processed food [57,58], which often has a high sugar or fat content and is known to increase risk of NCDs [59]. The risks of poor nutrition for young people during physical development are of particular concern, especially amongst disadvantaged people within LMICs [60]. With little influence over the nutritional transition occurring in their communities, there is an ongoing need for ways to address the impact of global trade structures [61].

Participants identified unevenly distributed health services throughout Vanuatu as a major barrier to health. In urban areas, long waiting times at clinics were cited as common, even for serious injuries such as breaks or fractures. In rural or remote areas, and small communities on distant islands, access to medical services were compounded by issues related to geography and low levels of economic development, creating interruptions to telecommunications and transportation. Moreover, participants in this study expressed a desire for improved communication between health workers and patients, and the ability to seek treatment without fear of judgement or prejudice. This is relevant for young teenage mothers, often victims of social stigma, whose demands for security and fair treatment have implications for improving maternal and child health [27].

There are multiple challenges faced by young people in Vanuatu that need to be overcome in order to bring about their desired level of health and well-being. The responses required both to alleviate threats to health and support increases in health service availability are complex, and capacity and finances are constrained. The participants in this research were clear in their suggestion that in Vanuatu no one sector is responsible, but that all levels of society are ultimately responsible for improving health throughout the nation’s complex geographical and social landscape.

The UN Development Programme (UNDP) has emphasised the importance of listening to Pacific youth voices on “how their islands should be developed once the MDGs come to an end in 2015.” Consequently, a series of debates were held among university students on Pacific perspectives on the post-2015 development agenda during 2012, in partnership with the University of the South Pacific and the Pacific Youth Council [62]. Although Diers [63] declared there was a clear need for post-2015 goals for adolescents around which UNICEF and others “can rally”, we generally found youth-related post-2015 activity was located within the more informal sphere of online blogs, social media commentary, and non-government organisation forums or reports.

Therefore, this study adds vital viewpoints of Vanuatu youth to the discourse on the post-2015 development goal agenda, and contributes to knowledge of youth health and development in LMICs [64]. This has far-reaching significance for ensuring a youth presence in dialogues of global importance [65], not to mention for health policy development more generally [66-68]. Youth recommendations require special attention, as this is the generation most likely to be greatly affected by the post-2015 agenda as it will apply throughout their reproductive and working years.

### *Study limitations*

While this study has attempted to include the perspectives of young people from diverse urban, rural and remote communities, a potential limitation of this study is the relatively small number of participants and its focus on perspectives of youth engaged in a secondary school environment, due to accessibility and resource constraints. This may be important given the low transition rate of students from primary to secondary education in Vanuatu [69]. Future research could include consultations with a wider cross section of youth not in school, working or unemployed. However, participants in this study have spoken of health issues experienced in common by others of their own age, which effectively broadens the reach of the study.

## Conclusion

Against a background of a weak health system and significant challenges to public health, young people are acutely aware that improving Vanuatu’s health status requires a communal effort. They recognize that while their health and well-being starts with the choices they make as individuals, ultimately their quest for achieving and maintaining good health encounters obstacles beyond their control. The youth in this study, unlike studies on youth health in high-income countries, focused on the link between the level of health attainable by individuals and the range of factors related to environmental and social determinants of health.

Furthermore, while contributing factors to health depend on actions taken at individual, local, national and even global levels, no single actor can provide enough support to cover all essential health needs. This reinforces the importance of including a youth voice and youth presence in policy making dialogue of the highest order. Health policy must address the barriers to this generation’s health across multiple sectors, including equitable access to community health education, national health system capacity, and structural influences resulting in the perceived unaffordability of locally grown produce versus cheap, unhealthy imported products made increasingly available due to the strength of the global food trade.

Participants endorsed an equitable approach to sustainable development in Vanuatu, as well as the need for all people locally and globally to take responsibility for a healthier world, in line with other post-2015 agenda discussion forums. Certainly, for this generation health in the Pacific is indeed “everybody’s role.” These are important considerations for the post-2015 development goal agenda and health policy makers in general.
